# Case report: Rudimentary uterine horn with ovarian endometriosis manifested as pelvic ectopic kidney

**DOI:** 10.3389/fmed.2023.1182355

**Published:** 2023-07-05

**Authors:** Si-fan Yin, Jia-gui Chai, Run-lin Feng, Zhi-yuan Yin, Shen-zhao Zhao, Tao Zhang, Chang-xing Ke

**Affiliations:** ^1^Department of Urology, The Second Affiliated Hospital of Kunming Medical University, Kunming, China; ^2^Department of Pathology, The Second Affiliated Hospital of Kunming Medical University, Kunming, China

**Keywords:** rudimentary uterine horn, endometriosis, pelvic ectopic kidney, diagnosis, treatment

## Abstract

**Background:**

Unicornuate uterus is a congenital uterine malformation. Unicornuate uterus with rudimentary horn, ovarian endometriosis, and congenital renal agenesis are rare combinations that can be easily misdiagnosed due to the lack of typical clinical manifestations.

**Case summary:**

A 19-year-old woman with pelvic pain was admitted to the hospital after a month. Physical examination was unremarkable. B-ultrasound and CT scan both indicated pelvic ectopic kidney. In addition, renal scintigraphy revealed normal perfusion and function of the right kidney, but the perfusion and function of the left kidney were not visible. A left pelvic ectopic kidney was diagnosed by preoperative images. A laparoscopic left pelvic ectopic nephrectomy was performed after adequate surgical preparation. However, the postoperative pathological diagnosis revealed a rudimentary uterine horn with ovarian endometriosis and congenital renal agenesis. Fortunately, she got recovered and was discharged from the hospital after 5 days following the operation. Moreover, she received regular follow-ups at the gynecology clinic. To date, no right adnexal or uterine abnormalities have been detected on ultrasound during the follow-up visits.

**Conclusion:**

Rudimentary uterine horn with ovarian endometriosis and congenital renal agenesis are rare and are easily Misdiagnosed due to the lack of typical clinical manifestations. A gynecological examination is recommended for patients who may have this disease.

## Introduction

Unicornuate uterus with a rudimentary horn is a rare congenital uterine malformation, whose clinical manifestations, including abdominal pain and dysmenorrheal, are atypical (1, 2). Moreover, imaging seldom yields an accurate result, which leads to diagnostic difficulties (3). In this article, we describe an unreported case of rudimentary uterine horn with ovarian endometriosis manifested as pelvic ectopic kidney to improve our understanding of the disease and thus the diagnosis and treatment of this disease.

## Case introduction

This patient was a 19-year-old G0P0 (gravidity = 0, parity = 0) woman. She was admitted to the hospital for pelvic pain after 1 month. No symptoms, such as frequent urination, urgent urination, painful urination, or hematuria, were noted.

**Figure 1:**
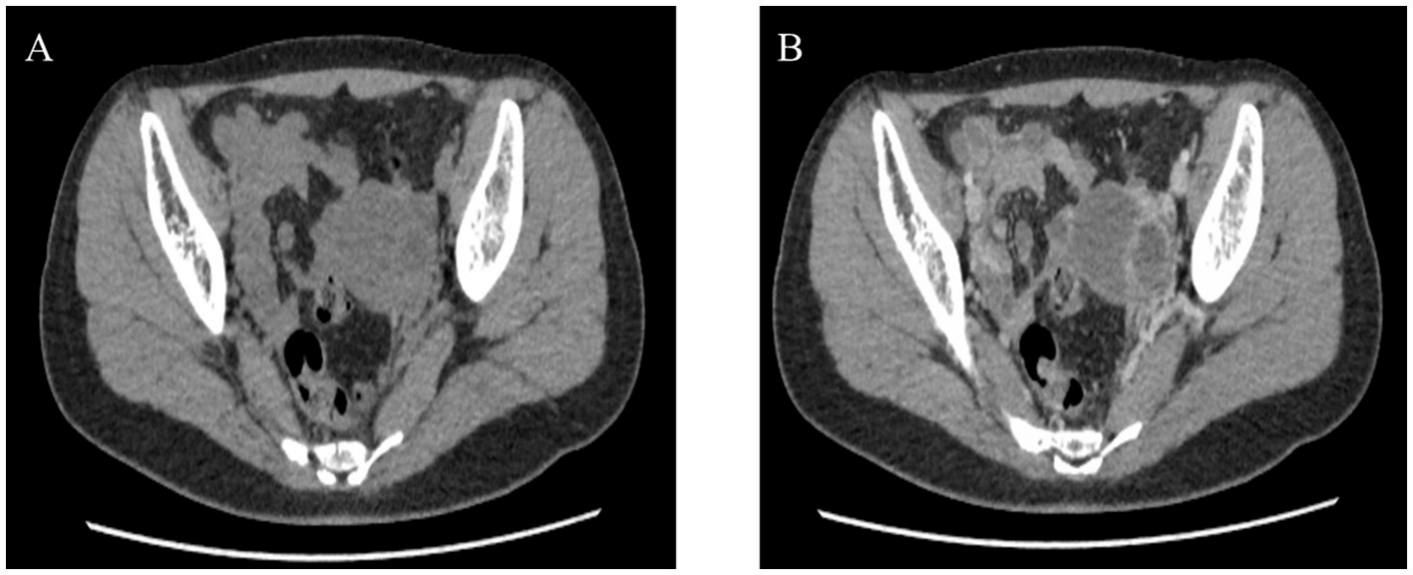
Preoperative CT, Heterotopic left kidney with congenital renal dysplasia and hydronephrosis; poorly visualized the left adnexa. **(A)** Plain CT. **(B)** Contrast-enhanced CT scan.

She had no specific history and denied any personal or family history associated with cancer. Physical examination revealed that no palpable masses and percussion pain were found in bilateral renal areas. Laboratory testing was unremarkable. An ultrasound scan showed no kidney in the left renal area. The pelvic cavity was detected as a heterogeneous structure (7.3 × 4.6 cm). The mass had a clear boundary and an irregular appearance, with punctate blood flow signals. A CT scan and enhancement showed that no renal was seen in the left renal area. A solid structure was seen in the left pelvis (3.35 × 3.7 cm). A lamellar, hypointense shadow is seen in the center, and the solid portion of the enhanced scan is clearly enhanced. A tortuous and thickened tubular structure was seen locally (Figure 1A). The enhanced scan showed marginal enhancement with a poorly visualized distal left ureter. The left adnexa was poorly visualized (Figure 1B). Imaging of renal dynamics: renal scintigraphy showed the right kidney to have normal perfusion and function, whereas the left kidney's perfusion was invisible. We combined this with preoperative imaging and diagnosed a left pelvic ectopic kidney.

**Figure 2:**
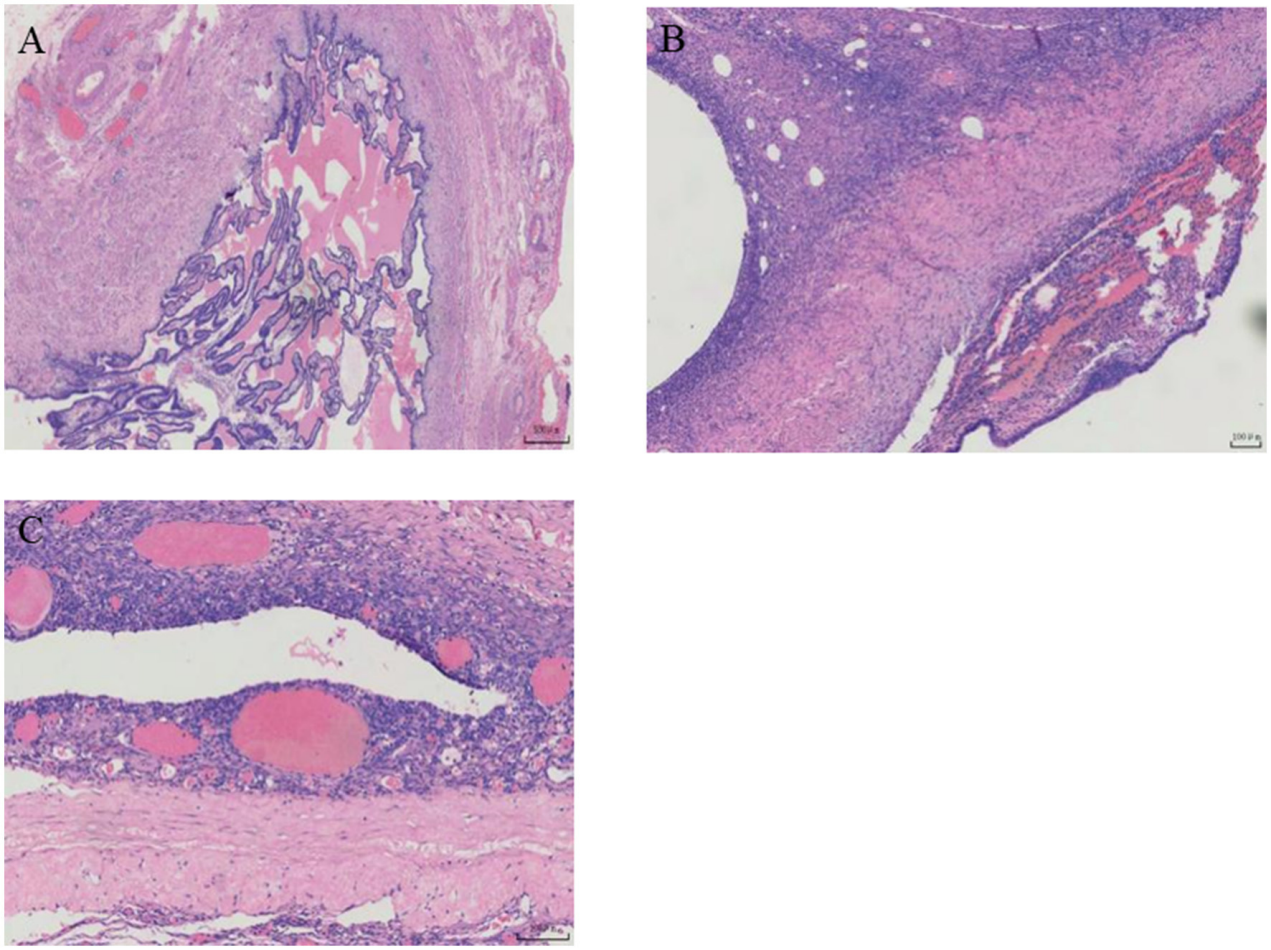
Postoperative pathological images. **(A, B)** Ovarianstroma with leukemogenesis and fallopian tube composition. **(C)** Ectopic endometrial epithelium and endometrial stromal components.

Our patient requested surgical treatment due to a non-functioning left kidney and pain. A laparoscopic left pelvic ectopic nephrectomy was performed after adequate surgical preparation. Intraoperatively, the uterus was visible in the middle. On the left side of the pelvis, a mass with apparent adhesion to surrounding tissues could be seen; despite consultation with a gynecologist, the ovary and fallopian tube were not identified. On the right side, the fallopian tube and ovary were observed to be normal. We diagnosed a unicornuate uterus combined with a left pelvic ectopic kidney intraoperatively by inviting a gynecologist for a consultation. After adequate separation of the mass, we removed them intact. Gross examination showed a grayish-white and grayish-brown mass with a size of approximately 6.5 × 4.5 × 3.5 cm. The cut surface was cystic, and dark red material was visible. Microscopy showed a scattered distribution of endometrial, ovarian, and fallopian tube components but no kidney components (Figures 2A–C). The pathological diagnosis was a rudimentary uterine horn with ovarian endometriosis and congenital renal agenesis.

**Figure 3:**
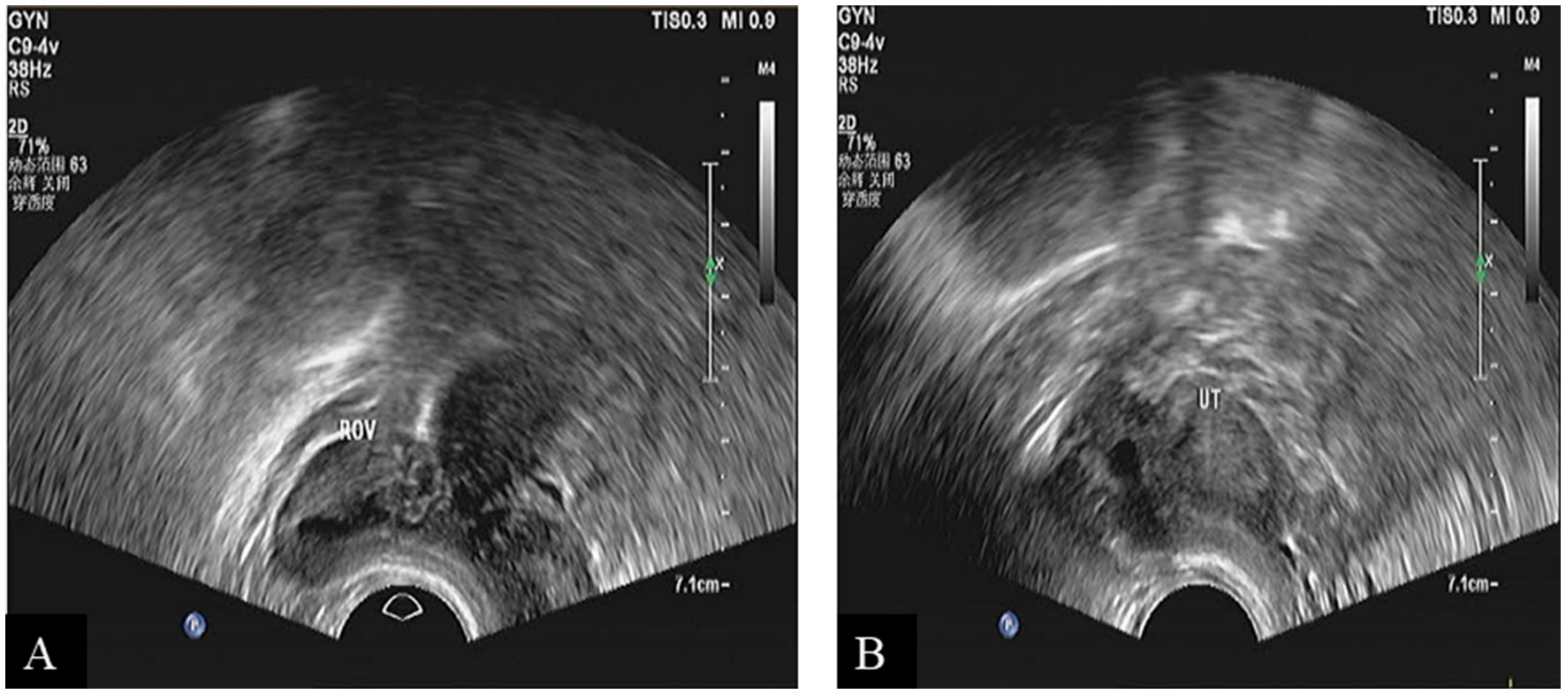
Ultrasound scan showed a unicornuate uterus during follow-up. **(A)** Right ovarian imaging. **(B)** Uterine imaging.

Fortunately, she recovered and was discharged from the hospital 5 days after surgery. Moreover, she received regular follow-ups at the gynecology clinic. To date, no right adnexal or uterine abnormalities have been detected on ultrasound during the follow-up visits (Figures 3A, B).

### Timeline

**Figure 4:**
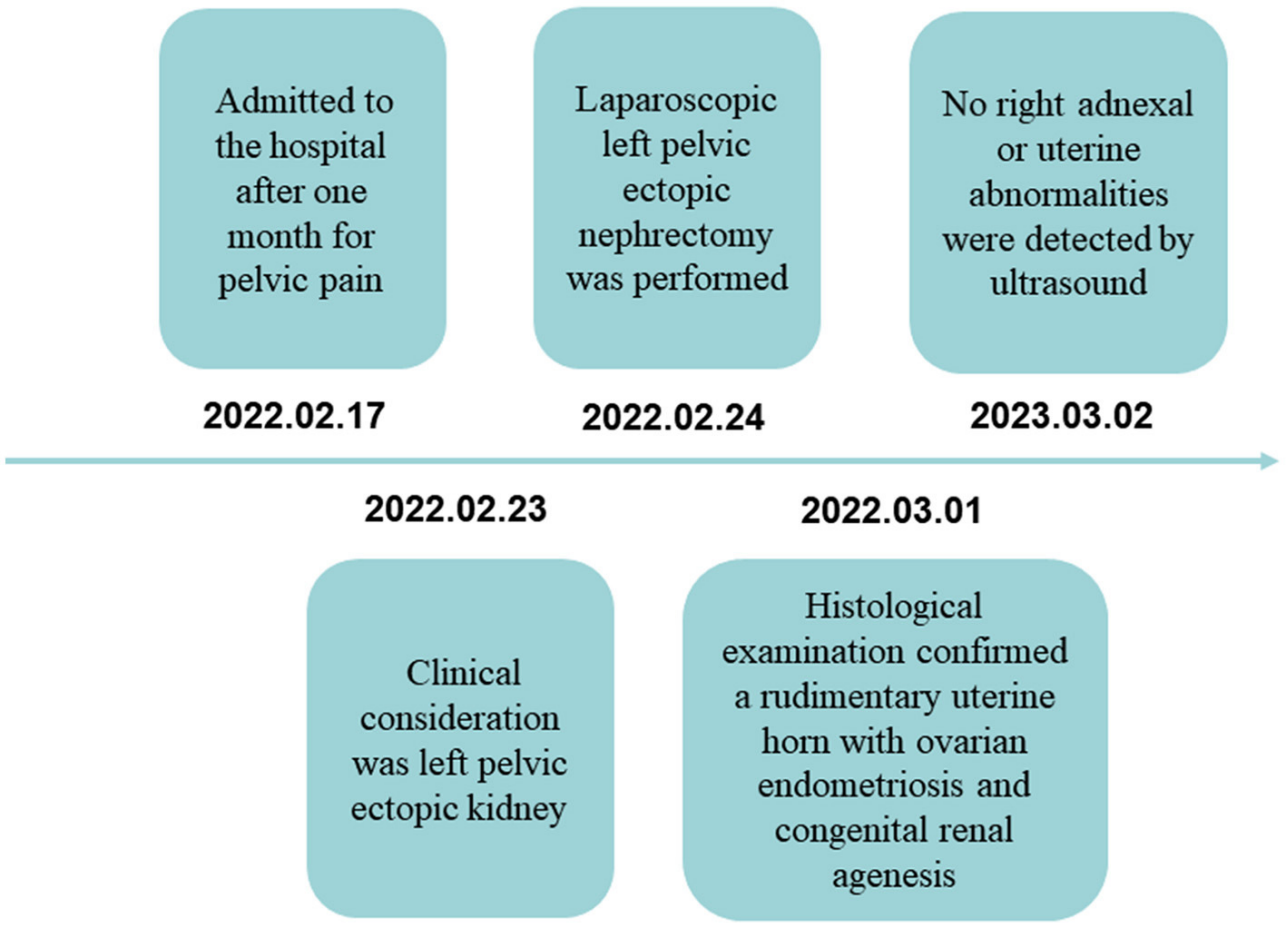
Timeline.

The patient was admitted to the hospital after one month for pelvic pain; relevant laboratory tests and examinations were completed, and clinical consideration was left pelvic ectopic kidney in the 6 hospital days; laparoscopic left pelvic ectopic nephrectomy was performed in 7 hospital days; definite histological examination confirming a rudimentary uterine horn with ovarian endometriosis and congenital renal agenesis was made in 12 hospital days. To date, no right adnexal or uterine abnormalities were detected by ultrasound at follow-up (Figure 4).

## Related literature learning

The incidence of unicornuate uterus is 1 in 4,000. Generally, the female reproductive system develops mainly from the Müllerian ducts. Müllerian ducts form the fallopian tubes, uterus, cervix, and upper two-thirds of the vagina, which together form the female reproductive tract. However, if one side of the Müllerian duct develops normally and the opposite side is defective, a unicornuate uterus is formed. According to the classification system of the American Society of Reproductive Medicine (ASRM) in 1988, this case is a type IIb uterine malformation, a unicornuate uterus with a non-communicating rudimentary horn (1, 4).

The unicornuate uterus is usually associated with urinary tract abnormalities because the urinary tract, gastrointestinal tract, and genitalia drain into a common chamber until the 5th week of embryonic development (5, 6). Statistically, the incidence of unicornuate uterus with renal hypoplasia or ectopic kidney is 15%, of which 40% are congenital renal agenesis on the side of the rudimentary horn (5, 7). In the present case, a rare unicornuate uterus with congenital renal agenesis on the rudimentary horn side was reported. In addition, a unicornuate uterus with ovarian endometriosis has also been reported.

Endometriosis is the occurrence, growth, and infiltration of endometrium-like tissue (glands and mesenchyme) in areas other than the endometrium. It may occur anywhere in the body, mainly in the pelvic organs, such as the ovaries (8, 9). The incidence of unicornuate uterus with endometriosis on the side of the rudimentary horn is 20~40%. The pathogenesis of endometriosis remains controversial. There are several theories, including retrograde menstruation, autoimmunity, coelomic metaplasia, and lymphatic and vascular metastasis, that attempt to explain the pathogenesis. According to the theory of retrograde menstruation, endometrial cells and tissues derived from menstruation retrograde implant into the abdominal cavity, invade and induce a local inflammatory response, which is accompanied by angiogenesis, adhesion, fibrosis, scarring, and anatomical distortion. Although most women have retrograde menstruation, not all of them have endometriosis. This suggested that affected women may have immune dysfunction that interferes with the cleaning of the lesions. Another theory is coelomic metaplasia which refers to the original celomic membrane undergoing metaplasia forming typical endometrial glands and stroma. Moreover, the theory of lymphatic and vascular metastasis suggests that the endometrium acts like neoplastic cells, invading the blood and lymphatic vessels and spreading elsewhere (5, 10, 11).

The pathogenesis of the present case was more likely explained by retrograde menstruation, in which menstrual debris containing viable endometrial cells refluxing through the fallopian tubes into the ovarium and pelvic cavity, leading to ovarian endometriosis and pelvic adhesion on the non-communicating rudimentary horn side of the patient (12).

The clinical presentations of rudimentary uterine horn with ovarian endometriosis, including dysmenorrhea and non-menstrual pelvic pain, are largely non-specific. It might be difficult to establish a definitive diagnosis in the early stages because of the non-specific and underlying symptoms, which can easily lead to severe consequences due to incorrect treatment (13).

Sonography of the unicornuate uterus does not provide a reliable diagnosis. Conventional 2-dimensional sonography is especially difficult to use for detecting the rudimentary horn because it cannot appreciate the small size and lateral deviation of an isolated unicornuate uterus. However, the use of 3-dimensional sonography improves diagnostic accuracy by obtaining reconstructed images that depict the deviation of the unicornuate uterus and the characteristic appearance of the endometrium. In addition, MRI allows accurate diagnosis of all subtypes of the unicornuate uterus due to its multiplanar imaging capabilities and excellent soft tissue resolution. Hysterosalpingography reveals an isolated unicornuate uterus with a small, fusiform uterine cavity. However, it is not recommended for routine application because of its invasive nature and inability to appreciate the non-communicating rudimentary horn (14).

In the present case, the clinical presentations of pelvic pain were non-specific. In addition, preoperative imaging showed no kidney in the left renal area. In contrast, a suspected renal mass was presented in the left pelvic region, so the diagnosis was considered a left pelvic ectopic kidney. There are differences in imaging between the rudimentary uterine horn and pelvic ectopic kidney (15). However, this case was misdiagnosed due to insignificant imaging differences, which may result from pelvic adhesion and inadequate gynecological examination.

The standard treatment option for the rudimentary uterine horn is surgical resection of the rudimentary horn and fallopian tube. However, confirmation of the diagnosis by adequate preoperative examination and intraoperative laparoscopic exploration is essential (5, 16). In the present case, despite an intraoperative consultation with the gynecologist, the ovary and fallopian tubes were not identified due to tissue adhesion, leading to an incorrect diagnosis and surgery. Therefore, detailed medical history collection (especially menstrual history) and preoperative gynecological examination are important for suspected congenital urinary abnormalities.

## Data availability statement

The original contributions presented in the study are included in the article/supplementary material, further inquiries can be directed to the corresponding authors.

## Ethics statement

Written informed consent was obtained from the individual(s) for the publication of any potentially identifiable images or data included in this article.

## Author contributions

S-fY, J-gC, TZ, and R-lF collected data and drafted the manuscript. R-lF provided figures and pathology results and drafted the manuscript. S-zZ and Z-yY reviewed the manuscript. C-xK edited the manuscript and critically revised the draft. All authors contributed to the article and approved the submitted version.
